# B lymphoblastic leukemia/lymphoma: new insights into genetics, molecular aberrations, subclassification and targeted therapy

**DOI:** 10.18632/oncotarget.19271

**Published:** 2017-07-15

**Authors:** Xiaohui Zhang, Prerna Rastogi, Bijal Shah, Ling Zhang

**Affiliations:** ^1^ Department of Hematopathology and Laboratory Medicine, H. Lee Moffitt Cancer Center and Research Institute, Tampa, Florida, USA; ^2^ Department of Pathology, University of Iowa College of Medicine, Iowa City, Iowa, USA; ^3^ Department of Hematological Malignancies, H. Lee Moffitt Cancer Center and Research Institute, Tampa, Florida, USA

**Keywords:** B lymphoblastic leukemia/lymphoma, molecular biology, genetics, prognostic markers, predictive markers

## Abstract

B lymphoblastic leukemia/lymphoma (B-ALL) is a clonal hematopoietic stem cell neoplasm derived from B-cell progenitors, which mostly occurs in children and adolescents and is regarded as one of top leading causes of death related to malignancies in this population. Despite the majority of patients with B-ALL have fairly good response to conventional chemotherapeutic interventions followed by hematopoietic stem cell transplant for the last decades, a subpopulation of patients show chemo-resistance and a high relapse rate. Adult B-ALL exhibits similar clinical course but worse prognosis in comparison to younger individuals. Ample evidences have shown that the clinical behavior, response rate and clinical outcome of B-ALL rely largely on its genetic and molecular profiles, such as the presence of *BCR-ABL1* fusion gene which is an independent negative prognostic predictor. New B-ALL subtypes have been recognized with recurrent genetic abnormalities, including B-ALL with intrachromosomal amplification of chromosome 21 (iAMP21), B-ALL with translocations involving tyrosine kinases or cytokine receptors (“BCR-ABL1-like ALL”). Genome-wide genetic profiling studies on B-ALL have extended our understanding of genomic landscape of B-ALL, and genetic mutations involved in various key pathways have been illustrated. These include *CRLF2* and *PAX5* alterations*, TP53, CREBBP* and *ERG* mutations, characteristic genetic aberrations in BCR-ABL1-like B-ALL and others. The review further provides new insights into clinical implication of the genetic aberrations in regard to targeted therapy development.

## INTRODUCTION

B lymphoblastic leukemia (B-ALL), a hematopoietic malignancy derived from B-cell progenitors, is predominantly a childhood disease but can occur in adolescents and adults as well. According to the 2016 United States statistics data of lymphoid neoplasm by World Health Organization classification, the incidence rate of B-ALL is 1.2% in 2011-2012 with an estimated 4930 new cases in 2016 [1]. It accounts for approximately 2% of the lymphoid neoplasms, and the incidence is approximately 11 cases per million persons per year in the United States [2]. Since 1980s, clinical outcomes of B-ALL patients have been steadily improved with an overall complete remission (CR) rate of >95% and 60-85% and overall survival (OS) rate of greater than 80% and less than 50% in children and adults, respectively [3–5]. The current treatment guideline recommends risk stratification based on patient age and *BCR-ABL1* translocation status. For example, patients with older ages, higher white blood cell counts, unfavorable cytogenetic changes, and residual disease after induction chemotherapy and comorbidities usually have a greater risk of relapse and shorter OS. The non-pediatric B-ALL patients are subclassified into four different groups based on age and *BCR-ABL1* status: 1) Philadelphia positive (Ph+) ALL adolescent and young adult (aged 15-39 years), 2) Ph+ ALL adult (aged ≥ 40 years), 3) Ph negative (Ph-) ALL adolescent and young adult, and 4) Ph- ALL adult [6]. Meanwhile, risk stratification of ALL in childhood is based on clinical and biological factors including age, white blood cell count, cytogenetics, response to initial induction chemotherapy, and involvement in central nervous system and testis [7]. Nevertheless, the current risk stratification system fails to identify a subgroup of refractory patients in low risk groups. The subset of patients with “low risk” behaves aggressively and could be undertreated without appropriate follow-up [8].

There have been major advances in recent years on the underlying pathogenesis of B-ALL, mostly attributed to the recent development of gene expression profiling and genome-wide sequencing analyses. In addition to revealing leukemogenesis of B-ALL in more depth, novel B-ALL subtypes with clinical significance have been proposed based on the newly emerged genetic data. Biomarkers with significant prognostic and predictive values (e.g., IKZF1, CRLF2, JAK, ABL1, ABL2, CSFR, PDGFRB, CREBBP) are identified. These markers would probably be, in part or in whole, integrated into the risk stratification system after validation through large clinical cohorts.

This review will summarize current understanding of B-ALL cytogenetics, and recently identified genetic aberrations, emphasizing on novel subclassification based on genetic changes, prognostic and predictive parameters that are directly related to clinical management of B-ALL patients.

## B-ALL CYTOGENTIC ABNORMALITTIES AND SUBCLASSIFICATION

**Figure 1:**
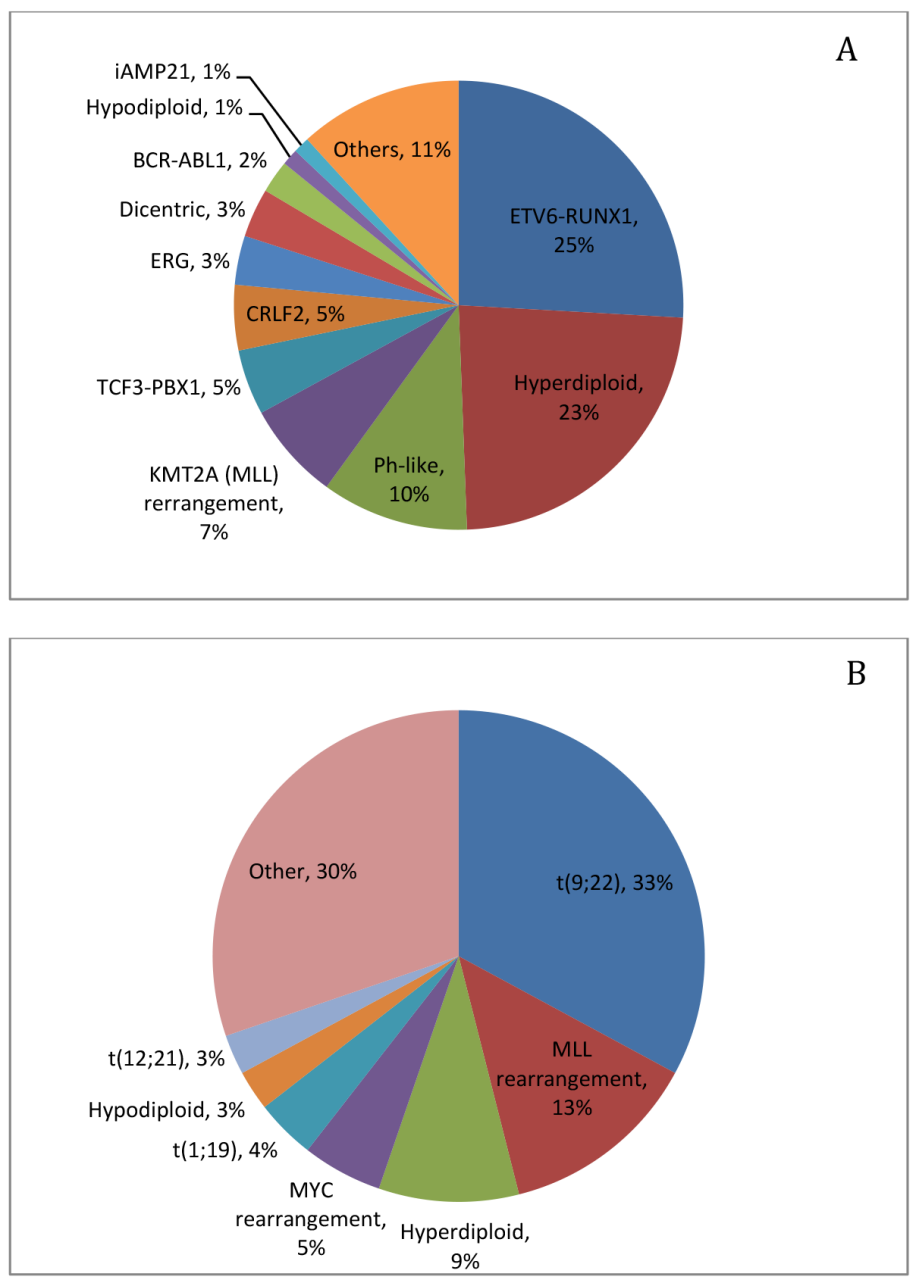
Frequency of cytogenetic and molecular genetic abnormalities in pediatric ALL **(A)** [9] and adult ALL **(B)** [12].

B-ALL is a heterogeneous disease that is associated with a plethora of chromosomal abnormalities, involving both numerical and structural alterations, such as hyperdiploidy, hypodiploidy, translocation, and intrachromosomal amplification. Approximately 75% of B-ALL cases have recurrent chromosomal changes detectable by conventional cytogenetic analysis [9], many of which have impacts on prognosis and are used for risk stratification on some treatment protocols [10] (Table 1, Figure 1).

**Table 1:** Common recurrent cytogenetic abnormalities in pediatric and adult B-ALL [3, 10, 11]

Risk groups	Cytogenetic abnormalities	Clinical significance	Frequency
Good risk	Hyperdiploidy (>50 chromosomes)	Favorable prognosis	25-30% in children; 7-8% in adults
	t(12;21)/ *ETV-RUNX1*	Favorable prognosis in children, undetermined in adults	25% in children; 0-4% in adults
Intermediate risk	t(1;19)/*E2A-PBX1*	Intermediate to favorable prognosis	1-6% in children; 1-3% in adults
	t(5;14)/*IL3-IGH*	Intermediate	Rare
Poor risk	t (9; 22)/*BCR-ABL1*	Poor prognosis	1-3% in children; 25-30% in adults
	t(v;11q23)/ *KMT2A (MLL)* rearrangements	Poor prognosis	2/3 in infants; 1-2% in older children; 4-9% in adults
	Hypodiploidy (<44 chromosomes)	Poor prognosis	6% in children, 7-8% in adults

Of note, two chromosomal abnormalities, including hyperdiploidy and t(12;21)/*ETV6-RUNX1* translocation, are associated with favorable clinical outcome. Cases with hyperdiploidy constitute one of the largest subgroups in B-ALL. The chromosomal gain is most often seen with chromosomes 4, 6, 10, 14, 17, 18, 21 and X [13] and least seen with chromosomes 1, 2 and 3 [14]. The overall prognosis is excellent [15]. However, extra number of specific chromosomes show different prognostic significance. For example, simultaneous trisomies of 4,10 and 17 carry the best prognosis [16]. Gain of chromosomes 4, 6, 10, and 17 indicates good prognosis [17], while gain of chromosome 5 or isochromosome 17 indicates poorer prognosis in this group [18]. Abnormality of t(12;21)/*ETV6-RUNX1* is usually cryptic by conventional karyotyping but detectable by fluorescence in situ hybridization (FISH) or polymerase chain reaction (PCR). The fusion protein likely acts in a dominant negative manner, disrupting normal function of the transcription factor RUNX1. Studies show that the translocation is an early event in leukemogenesis but by itself is insufficient for the development of overt leukemia [19]. Further cooperating genetic changes appear to be needed [20, 21].

Genetic abnormalities associated with an increased risk of disease relapse or worse prognosis include t(9;22) translocation (Philadelphia chromosome, or Ph chromosome), *KMT2A/MLL* translocations, and hypodiploidy. The t(9;22) translocation leads to a 190 kD or 210 kD BCR-ABL fusion protein, which is a dysfunctional tyrosine kinase. The incidence of t(9;22) increases with age and is present in up to 50% in older patients [22]. The clinical outcome with conventional chemotherapy in this patient group is extremely poor. However, tyrosine kinase inhibitors (TKIs) such as imatinib mesylate, in combination with intensive chemotherapy, have been used successfully, although primary or secondary drug resistance and high rates of relapse are problematic [23]. Mutations in *ABL1* (frequently T315I, Y253F/H, E255K/V, M351T, G250E, F359C/V, H396R/P, M244V, E355G, F317L, M237I, Q252H/R, D276G, L248V, F486S, etc.) are thought to be the major contributors to the drug resistance [24], for which new TKIs have been developed to bypass the signaling pathways or to bind to alternative sites, such as nilotinib, saracatinib, and ponatinib. They have shown great improvement on the clinical response in certain patients [25]. Nevertheless, clonal evolution, secondary gene aberrations such as deletions or mutations of *IKZF1* (discussed below) or other genes are found to be significantly associated with the resistance and relapse [26, 27]. Rearrangements involving the *KMT2A/MLL* gene located at chromosome 11q23 and one of the ∼80 fusion gene partners are most common in infants less than 1 year of age, and are associated with a poor prognosis. There is also a high frequency of central nervous system involvement at diagnosis. The most frequent partner gene is AF4, located at chromosome 4q21, with a fusion protein of *KMT2A/MLL*-AF4. The fusion proteins have an altered histone methylation pattern of *KMT2A/MLL* target genes, and subsequently cause leukemic transformation of the hematopoietic cells. Hypodiploidy (<46 chromosomes, with some people suggesting a stricter criteria with <44 chromosomes [28]) is a poor prognostic indicator. It can be further classified into different categories: high hypodiploidy (42-45 chromosomes), low hypodiploidy (33-39 chromosomes) and near haploidy (23-29 chromosomes) [29]. The patient has progressively poor prognosis with decreasing chromosome numbers. Near-haploidy and low hypoploidy B-ALL patients have extremely poor prognosis [30]. B-ALL with rearrangement of IGH locus occurs in less than 5% of the cases and confers poor prognosis [31]. The most common partner gene is cytokine receptor-like factor 2 (*CRLF2)* located at chromosome X, and other partner genes can be inhibitor of DNA binding 4 (*ID4*), *EPOR*, CCAAT/enhancer-binding protein (CEBP) family members, *BCL2*, the LIM domain homeobox 4 *(LHX4*) [17]. One of the rare B-ALL subtypes recognized in WHO classification is B-ALL with t(5;14) translocation which involves *IL3* and *IGH*, which is characteristically associated with non-clonal eosinophilia.

Some genetic changes that are associated with poor prognosis include the very rare t(17;19)/*E2A-HLF* translocation [32], abnormal 17p, and loss of 13q [10], as well as complex karyotype with 5 or more abnormalities in adult B-ALL patients [33]. Certain genetic changes that do not show significant impact on prognosis include t(1;19)/*E2A-PBX1* [34], del(6q), del(9p), and del(12p) [10, 11, 35, 36].

## NEWLY RECOGNIZED B-ALL SUBTYPES

In the 2016 revision of WHO classification, two new provisional B-ALL subtypes with recurrent genetic abnormalities have been recognized: B-ALL with intrachromosomal amplification of chromosome 21 (iAMP21), and B-ALL with translocations involving tyrosine kinases or cytokine receptors (“BCR-ABL1-like ALL”) [37]. These two entities further identify subgroups of B-ALL patients who have inferior clinical outcome and may benefit from more aggressive therapies or combination regimens with targeted therapy.

### B-ALL with intrachromosomal amplification of chromosome 21 (iAMP21)

Intrachromosomal amplification of chromosome 21 (iAMP21) is present in about 2% of pediatric B-ALL, mostly in older children and adolescents (median age 9 years), but is uncommon in adults. The patients harbor amplification of a large but variable region of chromosome 21, which can be detected by FISH with a *RUNX1* probe that reveals extra signals (5 or more copies per interphase nucleus, or 3 or more copies on a single abnormal chromosome 21 in metaphase FISH). This aberration manifests instability of chromosome 21 [38].

The patients are characterized by lower white blood cell and blast cell counts, older age, the French-American-British classification (FAB) L1 morphology, and common B-lymphoblast immunophenotype with a subset showing aberrant myeloid-associated antigen expression [39]. These cases can be detected by conventional karyotyping analysis by identification of the absence of a second normal copy of chromosome 21, which may not always be present, and concurrent FISH studies using *RUNX1* probe [39].

B-ALL with iAMP21 patients’ presentation of pancytopenia or mildly elevated white blood cell counts at diagnosis (usually ≤10 × 10^9^/L, with most ≤50 × 10^9^/L) may reduce the risk stratification. However, the patients demonstrated a consistently poor prognosis with worse event-free survival and OS when treated with standard-risk chemotherapy regimens [40]. In addition, cytogenetic change of iAMP21 has been confirmed to be a primary genetic event [38]. Therefore, B-ALL with iAMP21 is now considered as a distinct cytogenetic subgroup of B-ALL associated with a poor prognosis in pediatric patient population, and it is justified to assign such patients in the very high-risk group and treat them with more intensive chemotherapy. The clinical outcome has been significantly improved with more aggressive therapy [30, 41]. As iAMP21 is extremely rare in adults, its prognostic effect in adult group is undetermined.

### B-ALL with translocations involving tyrosine kinases or cytokine receptors (BCR-ABL1-like ALL, or Ph-like ALL)

BCR-ABL1-like ALL is a subgroup of B-ALL associated with unfavorable prognosis, which was originally described as a subgroup of childhood B-ALL that lacks chromosomal rearrangement of *BCR-ABL1* but exhibits similar gene expression profile to that of B-ALL with *BCR-ABL1* rearrangement [42, 43], and shares the similar poor prognosis and high risk for relapse [44, 45]. More studies suggest that BCR-ABL1-like ALL occurs in all age groups, accounting for up to 15% of children, 20-25% of adolescents and young adults, and 20-25% of adults with B-ALL, and is associated with event-free and OS rates equal or inferior to high-risk ALL subtypes, including BCR-ABL1 positive and *KMT2A/MLL*-rearranged B-ALL [8, 46, 47].

BCR-ABL1-like ALL is a heterogeneous subgroup involving many different genetic alterations. There are several common underlying genetic changes: translocations involving tyrosine kinases other than ABL1, translocation involving cytokine receptor genes such as *CRLF2*, and activating mutations or deletions of critical genes such as tyrosine kinase genes (*ABL1*, *JAK2*. etc.) and Ras signaling pathway genes [46] (see below for a more detailed discussion). More importantly, in this group, especially those with translocations involving tyrosine kinases, patients have improved clinical outcome with remarkable responses to TKI therapy [46, 48].

**Figure 2:**
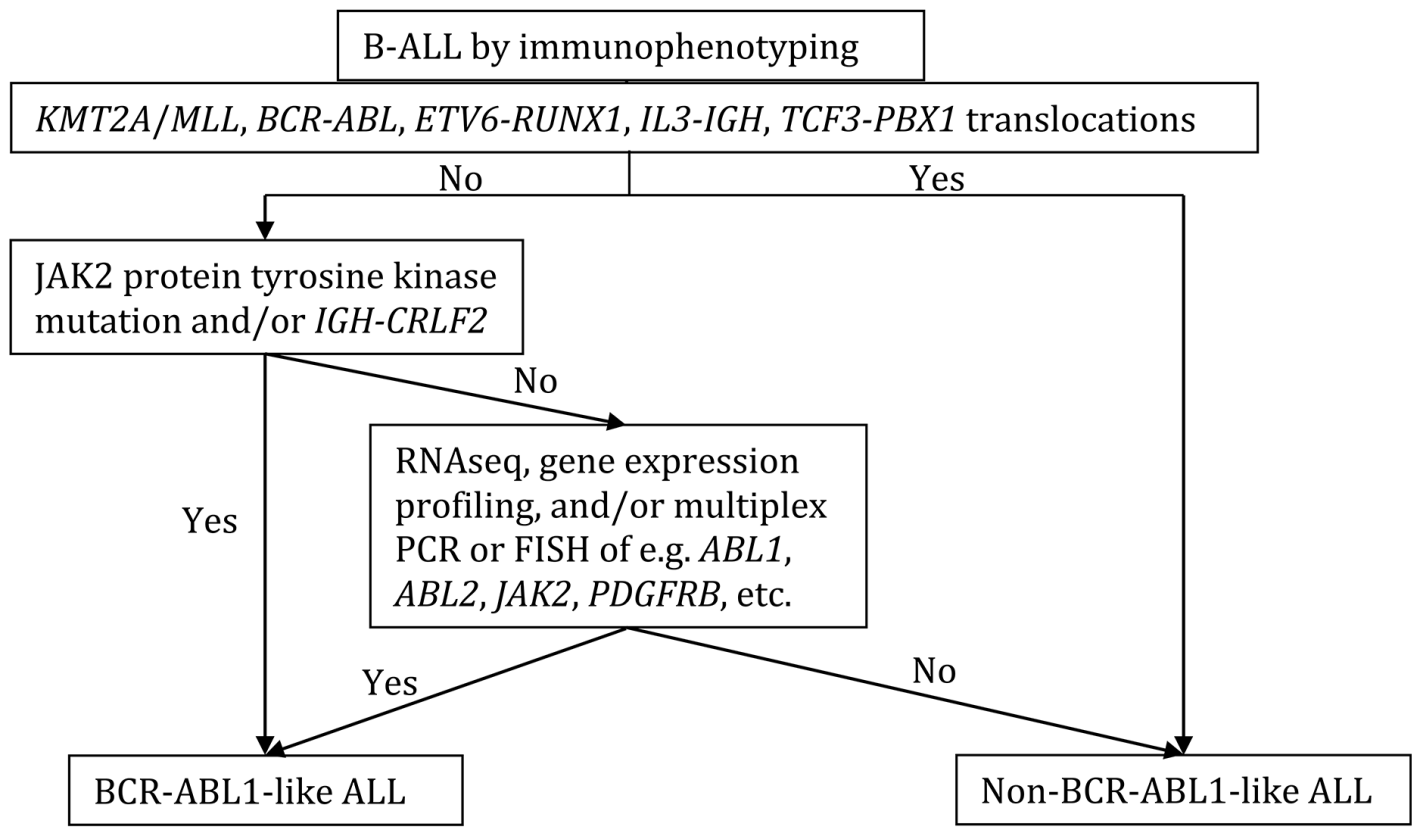
Proposed flow chart for the initial BCR-ABL1-like ALL workup Modified from [49].

Currently in the clinical setting it is difficult to identify such cases without gene expression profiling assays or genetic approaches such as genome and RNA sequencing, although certain laboratory screening assays have been used to identify some of the cases, such as targeted gene sequencing (RNA sequencing, RNAseq), low-density gene expression arrays, reverse transcription polymerase chain reaction (RT-PCR) and fluorescence in situ hybridization (FISH) for known translocations. Although there has been no standard guideline established for BCR-ABL1-like ALL diagnosis at initial workup of B-ALL, possible workflow was proposed, and is adapted in the following flow chart (Figure 2) [49].

## MOLECULAR GENETIC CHANGES

Genome-wide genetic profiling studies on B-ALL have extended our understanding of genetic landscape of B-ALL in children and young adults over the past decade. Mutations involved in various key pathways are found in different subtypes of B-ALL. The genes include transcriptional factors promoting early lymphoid cell development, e.g., *PAX5*, *IKZF1*, *EBF1*, *ETV6*, *LMO2,* which were detected in ∼40% of B-ALL cases [50], and other genes including tumor suppressor genes and cell cycle regulators (e.g., *TP53*, *RB1*, *CDKN2A/CDKN2B*)*,* cytokine receptor (e.g., *CRLF2, RPOR*), kinase (e.g., *ABL1*, *ABL2*, *CSF1R*, *JAK2*, *PDGFRB*), Ras signaling pathway (e.g., *KRAS*, *NF1*, *NRAS*, *PTPN11*), lymphoid signaling (e.g., *BTLA*, *CD200*), and epigenetic modification (e.g., *EZH2*, *CREBBP*, *SETD2*, *MLL2*, *NSD2*) [46, 50]. Among them, a few specific genetic alterations are found to be associated with adverse clinical outcome and increased risk for relapse [51]. Below are the most common molecular genetic changes identified in B-ALL, listed in a rough order of frequency reported in different B-ALL populations, and the most common genetic changes in BCR-ABL1-like B-ALL at the end.

### IKZF1 mutations

Mutations of transcription factors involved in early lymphoid development are considered a hallmark of B-ALL genetic changes. The transcription factors include *IKZF1*, *PAX5*, *EBF1*, *ETV6*, *LMO2, etc.* Among them, *IKZF1* mutation is one of the most frequent genetic aberrations in B-ALL. *IKZF1* gene encodes the Ikaros transcription factor that is an important regulator of normal lymphoid development and differentiation [52, 53]. *IKZF1* gene mutation is observed in high risk B-ALL, including approximately 80% of BCR-ABL1 positive B-ALL cases and 70% of BCR-ABL1-like B-ALL cases [54, 55]. *IKZF1* mutations are often deletions and rarely point mutations [56, 57]. Most deletions are monoallelic and involve exons 3-6, which encode the N-terminal zinc finger DNA-binding domain [56]. The deletions result in dominant negative form of the Ikaros protein that inhibits the function of wild-type Ikaros. It has been shown that induction of mutant, dominant negative Ikaros in early pre-B cells arrest the cell differentiation, suggesting that loss of Ikaros activity contributes to B-ALL leukemogenesis and *IKZF1* mutations are likely driver mutations [58]. Multiple studies support that *IKZF1* mutation/deletion is an independent indicator of B-ALL unfavorable clinical outcome including chemotherapy resistance and higher risk for relapse [27, 46, 59–61].

### CRLF2 alterations

*CRLF2* alterations are found in approximately 8% of pediatric B-ALL patients, and more than 50% of patients with Down-syndrome associated B-ALL [62]. *CRLF2* alterations are commonly gene rearrangement with immunoglobulin heavy chain locus resulting in *IGH-CRLF2* fusion gene, less often interstitial deletions resulting in *P2RY8*-*CRLF2* fusion gene, and rarely can be point mutations [63, 64]. These changes usually result in overexpression of CRLF2 (therefore can be analyzed by flow cytometry). CRLF2 alterations are associated with constitutive activation in the JAK-2 pathway such as JAK-STAT, PI3K/mTOR and BCL-2 transduction [65]. The alterations are often found in high-risk B-ALL [64], although the prognostic significance of CRLF2 deregulation in B-ALL remains controversial [63].

### PAX5 alterations

Alterations of *PAX5*, another key transcription factor involved in normal lymphoid development, have been found in ∼30% of B-ALL cases [50]. The alterations include acquired mutations, rearrangements involving various partner genes such as *ETV6* and *JAK2*, and germline mutations [50, 66, 67]. Unlike *IKZF1*, *PAX5* alterations do not appear to impact clinical outcomes, however, the *PAX5* mutations may be driver mutations in B-ALL leukemogenesis and play a role in susceptibility of B-ALL [67, 68].

### TP53 mutations

*TP53* deletions and mutations are initially found in 2-4% of pediatric patients [69] and 8% of adult patients [70] at initial diagnosis of B-ALL. However, next generation sequencing (NGS) data revealed that overall *TP53* mutations were present in up to 16% of B-ALL patients and the incidence increased with age and hypodiploid karyotype [71–73]. Notably, half of pediatric low hypodiploid B-ALL with 30-39 chromosomes show constitutional *TP53* mutations, indicating a unique association between low hypodiploid B-ALL and Li-Fraumeni syndrome [73]. Multiple studies suggest that TP53 aberrations at diagnosis are independently associated with early relapse and poor OS [74, 75].

### CREBBP mutations

Deletions and mutations of *CREBBP*, which encodes the transcriptional coactivators and acetyltransferase CREB binding protein, are found in 18% of relapsed pediatric B-ALL patients, but less than 1% at diagnosis in those who did not relapse [76], suggesting *CREBBP* gene mutations are associated with relapse of the disease. The mutations result in loss of function of CREBBP. In one study, *CREBBP* mutations were associated with hyperdiploid B-ALL relapse. Up to 60% of high-hyperdiploid relapse cases show CREBBP mutation, altering the clinical outcome in the favorable B-ALL group [77]. It might be a marker that can be integrated into risk stratification system after large cohort study.

### ERG mutations

Several studies have identified a subgroup of pediatric B-ALL patients, comprising 3-5% of B-ALL cases, with monoallelic deletion of *ERG* gene, which encodes an ETS-domain-containing transcription factor [78, 79]. The deletions result in an aberrant ERG protein that functions as a competitive inhibitor of wild-type *ERG* [80]. The *ERG* deletion and other known classifying genetic lesions are mutually exclusive, suggesting that B-ALL with *ERG* deletion may be a distinct subtype. Interestingly, these patients generally have excellent prognosis, despite an association with frequent *IKZF1* deletions, which is different from *BCR-ABL1* positive and *BCR-ABL1*-like B-ALL cases [79]. Whether or not the *ERG* mutations function as a negative regulator under *IKZF1* mutated status needs to be explored.

### Genetic aberrations in BCR-ABL1-like B-ALL

**Figure 3:**
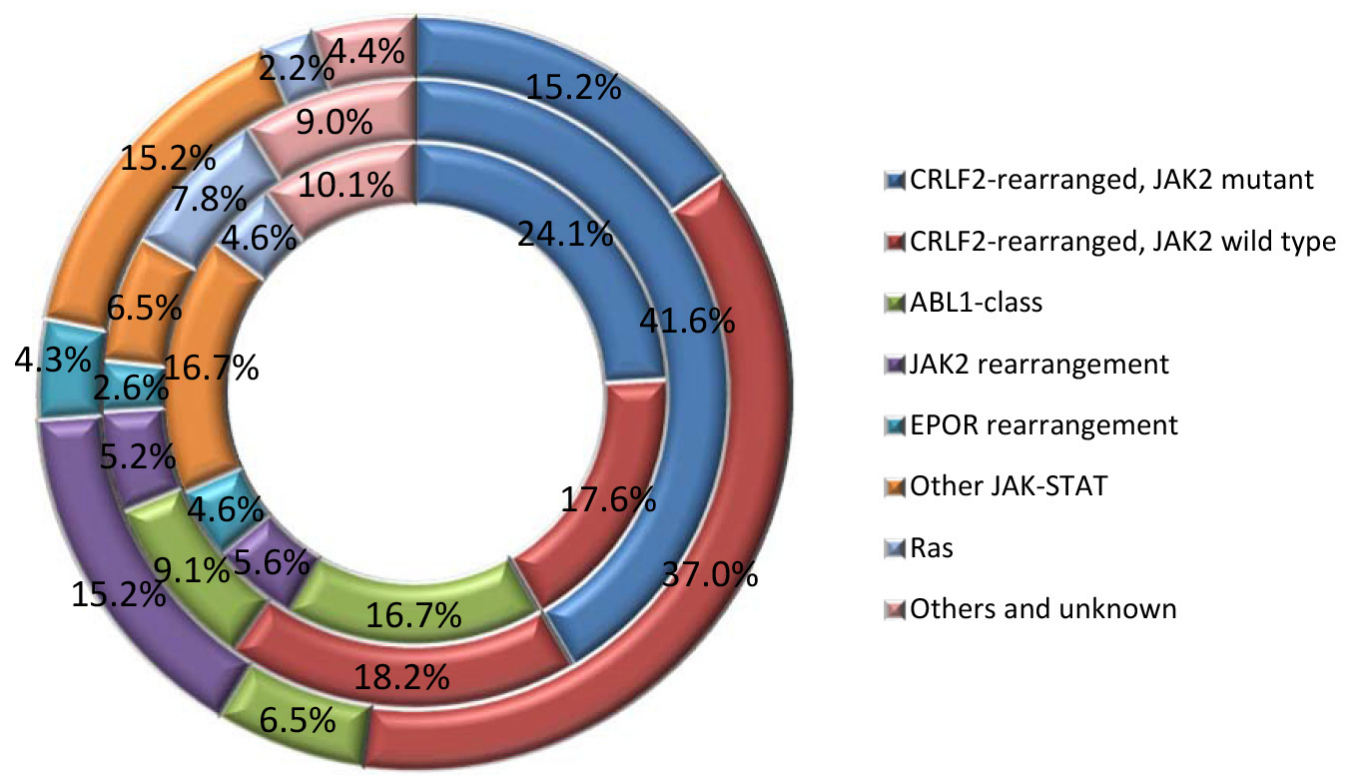
Breakdown of kinase alterations in children (inner doughnut), adolescents (middle doughnut) and young adults (outer doughnut) BCR-ABL1-like ALL [46, 84]

BCR-ABL1-like B-ALL is a unique group that is subcategorized under high risk B-ALL. The genetic abnormalities in this subtype of B-ALL involve a plethora of genes that can be categorized into different subgroups (Figure 3). They usually have a high frequency of *IKZF1* deletion (∼70%), CRLF2 overexpression (∼50%) and *JAK* mutations (∼30%) [46, 64]. Deletions or mutations of *IKZF1* are a hallmark of both BCR–ABL1–positive ALL as well as BCR-ABL1-like ALL [43, 50]. There are several types of kinase alteration in BCR-ABL1-like ALL including the rearrangements of *CRLF2*, rearrangements of *ABL* tyrosine kinase genes, rearrangements of *JAK2* and *EPOR*, mutations activating Janus kinase and signal transducer and activator of transcription (*JAK-STAT*) signaling and *Ras*, and less common kinase alterations (*NTRK3* and *PTK2B*) [81, 82]. Genomic profiling study on 154 B-ALL cases revealed most common rearrangements of kinase and cytokine receptor genes involving *JAK2*, *ABL1* (with partners other than *BCR*), and other genes controlling tyrosine kinases including *ABL2, CRLF2, CSF1R, EPOR, NTRK3, PDGFRB, PTK2B, TSLP, or TYK2*, and gene mutations involving *FLT3, IL7R*, or *SH2B*3 [46]. Notably, more than 80% of the BCR-ABL1-like cases have one or more genetic abnormalities in genes involved in B lymphoid cell development, including *IKZF1*, *TCF3 (E2A)*, *EBF1*, *PAX5*, and *VPREB1* [42]. Kinase activation and signaling via JAK-STAT and ABL-1 pathway are also considered key pathways in B-ALL leukemic transformation. Overexpression of the cytokine receptor CRLF2 was often found to have association with *JAK* mutations, especially *JAK2*.

In a brief summary, genetic changes underlying kinase-signaling pathways dysregulation include 1) *ABL1*-like rearrangements involving *ABL1, ABL2, CSF1R and PDGFRB*; 2) *JAK2* or *EPOR* rearrangements; 3) *CRLF2* rearrangements (often with *JAK* gene mutations and activation of JAK-STAT signaling); 4) Ras signaling pathway gene mutations; and 5) uncommon kinase alterations including *NTRK3, PTK2B TYK2*, etc. [46]. The clinical significance of the activation of kinase-signaling pathways is that the patients can be benefited from tyrosine kinase inhibitor therapy [46, 83].

## CLINICAL IMPLICATIONS OF GENETIC ABNORMALITIES

The current treatment regimens recommended by National Comprehensive Cancer Network guideline has mainly been based on risk stratification with appropriate chemotherapy intensification for high-risk patients [6]. Over the last several decades, advances in the treatment of B-ALL have significantly improved the clinical outcomes. Besides the successful utilization of TKIs in the treatment of BCR-ABL1-positive B-ALL, collaborative studies such as the project Therapeutically Applicable Research to Generate Effective Treatments (TARGET) (https://ocg.cancer.gov/programs/target/) use comprehensive molecular characterization to determine the genetic changes and identify therapeutic targets and prognostic markers. The studies identified novel targets for therapy in high risk and relapsed B-ALL, as summarized in Table 2 [85].

**Table 2:** Potential targeted therapy in B-ALL ([46, 85], if not otherwise specified)

Altered singling pathways	Inhibitor	FDA approved medication	Potential agents
BCR-ABL1-like ALL			
ABL1, ABL2, CSFR, PDGFRB	TKIs	Imatinib * Dasatinib* Ponatinib*	
CRLF2, JAK2, EPOR, TSLP	JAK2 inhibitor	*Ruxolitinib #*	
IL2RB	JAK1/JAK3 inhibitor	*Tofacitinib #* *Oclacitinib #*	
NTRK3	NTRK3 inhibitor	*Crizotinib #*	
TYK2	TYK2 inhibitor		Ndi-031301 [92]
PTK2B	FAK inhibitor		VS-4718 [93]
CREBBP (CREB-binding protein- CBP)	Histone deacetylase (HiDAC) inhibitors		ICG-001 (bind to CBP) [94]
Mutations in Ras/RTK pathway and PI3K pathway genes	PI3K/mTOR inhibitors	*Rapamycin #* [95]	
*MLL/KTM2A* rearrangement	Inhibitor of histone methyltransferase: DOLT1, FLT3 inhibitors	*Lestaurtinib #* (FLT-3 inhibitor) [96, 97]	
Hypodiploidy (TP53, RAS/RTK/PI3K pathways)	MEK inhibitors PI3K inhibitors	*Trametinib #* [91]	Selumetinib [90]
Hyperdiploidy (RAS pathway)	MEK inhibitors	*Trametinib* # [91, 98]	Selumetinib [90]

*. FDA approved for lymphoblastic leukemia treatment

#. FDA approved for other diseases but not for acute lymphoblastic leukemia

TKIs addition to cytotoxic chemotherapy in patients with BCR-ABL1-like B-ALL has significantly improved even-free survival and OS [46, 48, 83, 86]. The group of TKIs has been widely implicated in B-ALL patients who harbored *ABL1, CSF1R* and *PDGFRB* aberrations. Studies have shown that BCR-ABL1-like B-ALL cases, including cases with gene rearrangement involving *ABL1, JAK2, PDGFRB* and *IL7R* and other tyrosine kinase genes, had a poor response to conventional induction chemotherapy but showed sustained responses to TKIs [46, 48, 83, 86].

Preclinical studies demonstrated that JAK kinase inhibitors can be used in B-ALL with activated JAK-STAT signaling such as in B-ALL with *CRLF2* rearrangements and *JAK* gene mutations [87]. JAK2 inhibitors are particularly used in the subgroup of B-ALL with *JAK2, EPOR, CRLF2* and *TSLP* aberrations*,* while JAK1 and JAK3 inhibitors selectively inhibit patients with *IL2RB* gene rearrangements. Likewise, PI3K/mTOR pathway inhibitors and Ras signaling pathway inhibitors are exploited and may become new therapeutic targets for high risk and relapsed B-ALL subgroups [88, 89]. Multiple small molecules developed to target specific pathways in the preclinical studies have shown potential treatment effects as well. MEK inhibitors (e.g. selumetinib) can overcome glucocorticosteroid resistance in B-ALL [90]. Korfi K. et al. used anti-MEK molecule, MEKi/Trametinib, and BCL-2/BCL-XL family inhibitors to increase inhibitory functions and to induce apoptosis in B-ALL cells [91].

Besides the aforementioned targeted therapies for newly diagnosed or refractory B-ALL, several monoclonal antibodies (anti-CD20, rituximab; anti-CD52, alemtuzumab; and anti-CD22, epratuzumab) are developed and added to conventional chemotherapy to achieve optimal clinical outcomes. Novel agents such as inotuzumab ozogamicin (anti-CD22, immunoconjugate) and blinatumomab (anti-CD19 BiTE antibody) have also been adopted as single agent therapy for those with relapsed or refractory B-ALL [99]. Clinically a combination of monoclonal antibody and targeted pathway inhibitor might be the option to achieve synergistic effects. In addition, targeted immunotherapty using chimeric antigen receptor (CAR) modified T cells targeting CD19 has emerged as a powerful targeted immunotherapy, particularly to relapsed and refractory B-ALL with high response rates and durable remissions reported [100].

However, it is still in early stage for B-ALL target therapy, and it may take a lengthy time to translate the promising therapeutic agents in preclinical studies to clinical implementation. Development of more inhibitory small molecules, study of their efficacy and side effects clinically and careful clinical evaluation of the long term outcome of the targeted agents, including combination use of two or more agents, are the future directions. It will be necessary to put more priority on treatment of high-risk and relapsed B-ALL cases, and to minimize development of drug resistance.

## CONCLUSIONS

In conclusion, gene expression profiling and genome-wide sequencing analyses have made great advancement over the past few years in understanding B-ALL biology and genetics. The development is very helpful in subclassifying B-ALL patients with different risks, identifying novel therapeutic targets, and improving the overall clinical outcomes. Biomarkers with prognostic and predictive values, as well as targeted therapeutic agents, have been emerged as promising approaches in clinical care of B-ALL in the era of personalized medicine.

Still, current challenges include fully understanding the genetic basis of B-ALL, discovering more efficacious therapeutic regimens, and importantly, identifying B-ALL subgroups with characteristic molecular features that can be used in targeted therapy, such as B-ALL with other kinase-activating aberrancies. There is a need to implement molecular diagnosis and subclassification in our practice, in order to utilize the potentially more efficacious therapeutic agents for this malignancy in the era of personalized medicine.
